# How to Preserve Health

**Published:** 1890-08

**Authors:** 


HOW TO PRESERVE HEALTH. By LOUIS BARKAN, M. D American News Co., New York. Pp. 344.—Paper. Bound in cloth, $1.00.

This book, written for lay readers, is full of useful knowledge. It is fully abreast of the times, and, unlike so many books of its class, it does not attempt to make doctors out of its readers.

In addition to hints on hygiene, it devotes a goodly portion of its pages to nursing special diseases.

We cheerfully recommend this publication. J. W. P.

